# HSP105 expression in cutaneous malignant melanoma: Correlation with clinicopathological characteristics

**DOI:** 10.1371/journal.pone.0258053

**Published:** 2021-10-07

**Authors:** Ke-Jun Chen, Feng-Zeng Li, Qian Ye, Meng Jia, Sheng Fang

**Affiliations:** Department of Dermatology, The First Affiliated Hospital of Chongqing Medical University, Chongqing, China; Virginia Commonwealth University, UNITED STATES

## Abstract

**Background:**

Heat shock proteins can protect against stress-associated cellular challenges, but they can also protect some tumors from human immune system monitoring. Heat shock protein 105 (HSP105/110) is a high molecular weight protein whose expression has been reported in many cancers, but few studies on its role in cutaneous malignant melanoma have been published. In this study, we analyzed the relationship between HSP105 expression and the clinicopathological characteristics of CMM.

**Methods:**

This retrospective study included 91 patients with CMM. The clinicopathological characteristics of CMM patients, including age, lesion duration, location, pathological classification, Clark’s level, Breslow thickness, metastasis and recurrence, were collected. Immunohistochemical staining and Western blot analysis for HSP105 were performed. Pigmented nevi (n = 20) served as a control. The staining intensity and percentage of stained cells were expressed as a histochemical score (HSCORE).

**Results:**

HSP105 was overexpressed in melanoma compared with nevi. Differences in the HSCORE between nevi (HSCORE = 1.05(0.15,1.50)) and CMM (HSCORE = 2.68(1.80,3.60)) were remarkable (P<0.001). Exposed site lesions, recurrent and metastatic lesions, nodular melanoma and lentigo maligna melanoma were closely associated with higher HSP105 expression (P = 0.011, P = 0.001 and P = 0.001, respectively). Moreover, no significant difference was observed in Clark’s level, Breslow thickness, or lesion duration (P>0.05).

**Conclusion:**

HSP105 is overexpressed in CMM. Higher HSP105 expression in lesions is associated with different clinicopathological variables. HSP105 may be a potential target for the diagnosis, treatment and prognostic prediction of CMM.

## Introduction

Heat shock proteins (HSPs) are highly conserved proteins present in the cells of all organisms. HSPs act as chaperones to protect proteins from destruction and to help repair misfolded proteins. In most normal situations, the expression of HSPs is low. However, HSPs will be expressed to protect the body and improve the cell stress response in response to stressors [1]. Proteins in the HSP family can be divided into five groups according to their molecular weight: small molecule HSPs, HSP40, HSP70, HSP90, and HSP105/HSP110. HSP105/110 is a major mammalian HSP that differs from the HSP70 family [2]. Overexpression of HSPs has been found in a wide range of cancers; therefore, diverse diagnostic and treatment methods are under investigation [3].

Cutaneous malignant melanoma (CMM) is the most invasive and fatal form of skin cancer that has a high fatality rate once metastasis occurs [4]. Currently, various treatment approaches, such as surgical resection, human interferon-beta, targeted therapy and immunotherapy, are used. Up to 90% of melanomas exhibit aberrant MAPK (Ras/RAF/MEK/ERK) pathway activation, and approximately 80‑90% of BRAF mutations are the V600E (valine to glutamic acid) mutation. PI3K/AKT is the second most frequently activated pathway in melanoma [5]. Previous studies have shown that HSP105 is closely related to melanoma, and evidence from mouse models has shown that the HSP105 DNA vaccine can induce activation of CD4+ T cells and CD8+ T cells, which play important roles in antitumor immunity [6]. Most malignant melanomas exhibit increased expression of iASPP (inhibitor of an apoptosis-stimulating protein of p53) [7], while HSP105 can bind to the tumor suppressor gene p53 to protect cells from apoptosis, which is essential for the survival and proliferation of cancer cells [8–10]. HSP105 was shown to be overexpressed in a variety of human cancer cells, and high expression of HSP105 in squamous cell carcinoma [11], lung adenocarcinoma [12] and oral squamous cell carcinoma [13] is always related to disease progression and poor prognosis. Although several studies have revealed that HSP105 expression in CMM and metastatic CMM is higher than that in nevi [14], the relationship between HSP105 and clinicopathological features of CMM has not been identified. Therefore, we examined the level of HSP105 expression in CMM and nevi and evaluated the relationship between HSP105 and the clinicopathological characteristics of CMM. These results are beneficial for understanding the involvement of HSP105 in the pathogenesis of CMM and provide a basis for future targeted therapy against HSP105 according to clinicopathological features.

## Materials and methods

### Participants

We conduct a retrospective analysis for a total of 91 paraffin-embedded CMM tissue samples provided by the department of dermatology of the First Affiliated Hospital of Chongqing Medical University in 2021. The samples represented CMM patients who underwent surgical resection between January 2016 and June 2021. We also collected clinicopathological data from these patients’ medical charts and medical records system. The 20 paraffin-embedded nevus tissue samples were used as control in the same period. None of the patients received radiotherapy or chemotherapy before surgery. The clinicopathological data of 91 patients with CMM were collected, including sex, age, lesion duration, location, clinical stage, recurrence, and pathological classification. We divided the locations into exposed sites (head, face, neck, V-neck, outer forearms and dorsum of the hand) and non-exposed sites.

### Ethics approval and consent to participate

This study was approved by the Medical Ethics Committee, the First Affiliated Hospital of Chongqing Medical University (Reference number: 2020–889). Our analysis was a retrospective design using fully anonymized data, and thus, the IRB waived the requirement for informed consent.

### Immunohistochemistry

Paraffin sections (4 μm) were prepared, deparaffinized by dipping in xylene for 10 minutes and rehydrated through a descending alcohol series to distilled water. The sections were then boiled for 20 minutes at 100°C in citrate antigen retrieval buffer and then cooled to room temperature. Endogenous peroxidase activity was blocked by treatment with 3% hydrogen peroxide in methanol for 5 minutes, while nonspecific binding was blocked by incubation with 3% BSA in PBS at room temperature for 30 minutes. Tissue sections were then incubated overnight at 4°C with an HSP105 antibody (1:200 dilution, 109624, Abcam, Cambridge, UK). After several washes in PBST (0.05% Triton X-100 in PBS), the slides were incubated with biotinylated goat anti-rabbit IgG (Vector Laboratories, Burlingame, CA) at room temperature for 30 minutes. After a further wash in PBST, streptavidin–horseradish peroxidase was added, and horseradish peroxidase activity was detected with a DAB peroxidase substrate kit (Vector Laboratories, Burlingame, CA). Finally, sections were counterstained in hematoxylin, followed by dehydration in graded alcohol solutions, dipping in xylene, neutral resin sealing, and air-drying. Negative control sections included in each staining experiment were prepared using rabbit immunoglobulin (isotype: IgG1, concentration: 100 μg/mL;) diluted at 1:15,000. Finally, we observed sections using a pathology scanner (KFBIO, model: KF-PRO-005).

### Western blotting

Tissue samples for Western blot analysis were frozen in liquid nitrogen soon after excision and stored at − 80°C until use. The specimens were either CMM or nevi, and patients provided informed consent before surgery. Patient tissues were obtained in accordance with the guidelines and approval. Briefly, tissues were lysed in cold RIPA buffer in the presence of protease/phosphatase inhibitors (Sigma-Aldrich, St. Louis, MO). After centrifugation to remove precipitate, the protein concentration was quantified using the bicinchoninic acid assay (Thermo Scientific, 23225). Normalized lysates were then mixed with sample loading buffer containing 2-mercaptoethanol and SDS and heated for 10 minutes at 100°C. A total of 40 μg of normalized total proteins from lysates was run on 10% SDS-polyacrylamide gels. The proteins were transferred from gels to nitrocellulose membranes (Millipore, Billerica, MA, USA), which were incubated with HSP105 antibodies (1:1000 dilution, 109624, Abcam, Cambridge, UK) overnight at 4°C, washed, and incubated with HRP-labeled goat anti-rabbit IgG (H+L) (Beyotime, A0208) for 1 h at room temperature. Visual signals were detected using the BIO-RAD ChemiDoc XRS Imaging System.

### Evaluation of HSP105 expression

To avoid bias, all findings were evaluated by three independent pathologists who were blinded to the patients’ clinicopathological data. Immunohistochemical staining data were expressed as the histochemical score (HSCORE) based on the intensity and percentage of stained tumor cells according to the following equation: HSCORE = ∑Pi(i +1), where i is the intensity of staining with a value of 0, 1, 2 or 3 (score 0 (absent), score 1 (weak), score 2 (moderate), score 3 (intense)) and Pi is the percentage of stained tumor cells varying from 0% to 100% (0.0–1.00). HSCORE values ranged from a minimum of 0 in cases with no staining to a maximum of 4.0 in cases where all the tumor cells were stained with maximal intensity. Due to the abnormal distribution in our data, the median (M) and upper and lower quartiles (P25, P75) were used to describe the HSCORE.

### Statistical analysis

HSP105 expression was compared using the nonparametric Mann–Whitney (nevus and CMM, tumor location, metastasis and recurrence) and Kruskal–Wallis (pathological classification) tests, as well as Spearman rank correlation tests (duration of the lesion, Clark’s level and Breslow thickness). Statistical analysis was performed using SPSS software for Windows (version 25.0; SPSS Inc.). Statistical significance was set at P <0.05 throughout the study.

## Results

### General data

The general nevus and CMM data are presented in Table 1. In all, 91 patients with CMM and 20 patients with nevi were identified. Among the nevus patients, 12 (60.0%) were male and 8 (40.0%) were female. The average age was 42 years (range 23–69 years). This group included junctional nevus (5 cases), intradermal nevus (8 cases), and compound nevus (7 cases). In most cases, pigmented nevi were located on the head/face/neck (5 cases) and lower limbs (7 cases), whereas the trunk and upper limb were rarer sites. Of the patients with CMM, men (52.8%) were affected more frequently than women (47.3%). The average age was 59.75 years (range 26–84 years). Most CMM tumors were located in the lower extremities (35.2%), whereas the number of cases of melanoma on the face and neck was relatively small (16.5%). Twenty-nine patients had acral lentiginous melanoma. In addition, other variants included lentigo maligna melanoma (17 cases), nodular melanoma (24 cases) and superficial spreading melanoma (21 cases).

**Table 1:** Clinicopathological features of Nevus and CMM.

Characteristics	Nevus N (%)	CMM N (%)
**Overall number**	20	91
**Gender**		
Male	12 (60.0)	48(52.7)
Female	8 (40.0)	43(47.3)
**Age**		
(Median, mean, range)	(42y,42y,23-69y)	(57y,59y,26-84y)
**Location**		
Head/Face/Neck	5 (25.0)	15 (16.5)
upper limb	3 (15.0)	19 (20.9)
lower limbs	7 (35.0)	32 (35.2)
Trunk	2 (10.0)	25 (27.4)
**Pathological Classification**		
	Junctional 5(25.0)	LMM 17 (18.7)
	Intradermal 8(40.0)	NM 24 (26.4)
	Compound 7(35.0)	SSM 21 (23.1)
		ALM 29 (31.8)

CMM, cutaneous malignant melanoma; ALM, acral lentiginous melanoma; NM, nodular melanoma; LMM, lentigo maligna melanoma; SSM, superficial spreading melanoma.

### Expression of HSP105 in nevi and CMM

**Fig 1:**
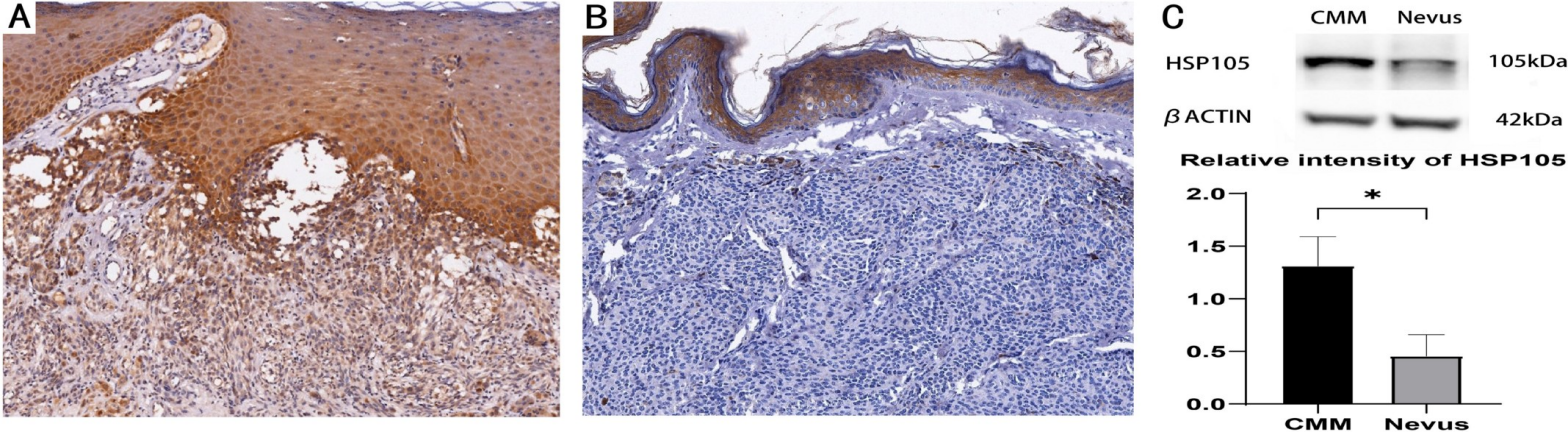
HSP105 expression in CMM and nevus. (A) HSP105 expression in CMM with intense staining (IHC, ×100). (B) HSP105 expression in Nevus with weaker staining (IHC, ×100). (C) The Western blot analysis of HSP 105 expression in CMM and Nevus. Comparison was showed in the column graph. Values represent the mean ± SD.*, P < 0.05.

To explore the significance of HSP105, a control group of 20 benign pigmented nevi was used to evaluate whether HSP105 expression was increased in CMM. Both melanoma and pigmented nevus shows intense staining in epidermis. The immunohistochemical staining pattern of HSP105 in CMMs and nevi was examined (Fig 1). HSP105 staining in CMM cells was intense (Fig 1A) but was absent (Fig 1B) in nevus cells. Differences in the HSCORE between nevi (HSCORE = 1.05(0.15,1.50)) and CMM (HSCORE = 2.68(1.80,3.60)) were remarkable (P<0.001). The results were consistent with those of the Western blot analysis, which showed increased HSP105 expression in melanoma compared with nevi (P<0.05) (Fig 1C).

### The relationship between HSP105 and clinical features

**Fig 2:**
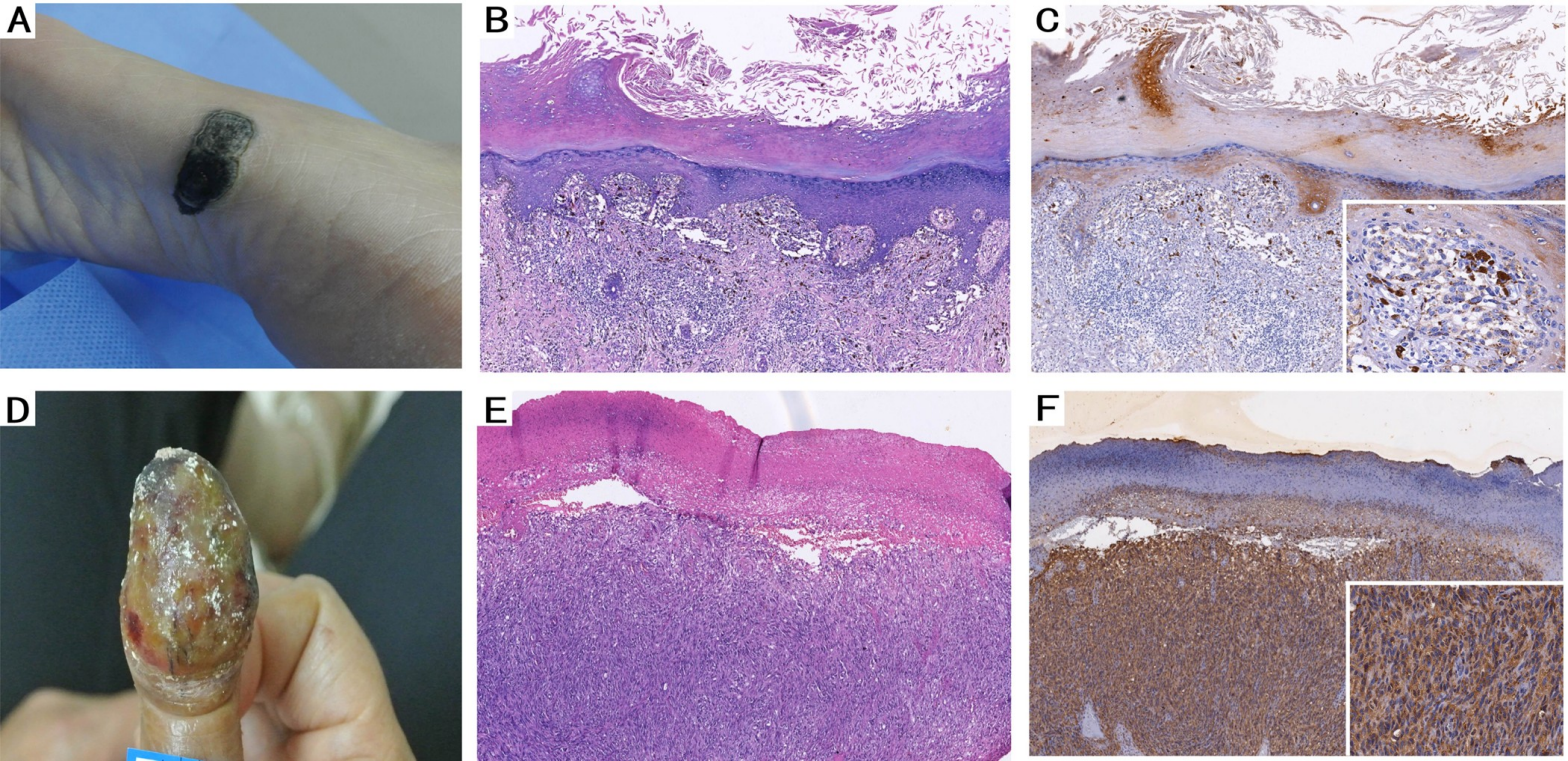
HSP105 expression in non-exposed site and exposed site. (A) Clinical picture of the LMM located in foot (non-exposed site). (B) Histological appearance (HE, ×100) in the LMM located in non-exposed site and (C) HSP 105 expression showed focal staining (IHC×100). (D) Clinical picture of the LMM located in hand (exposed site). (E) Histological appearance (HE, ×100) in the LMM located in exposed site and (F) HSP 105 expression showed intense staining (IHC×100).

**Fig 3:**
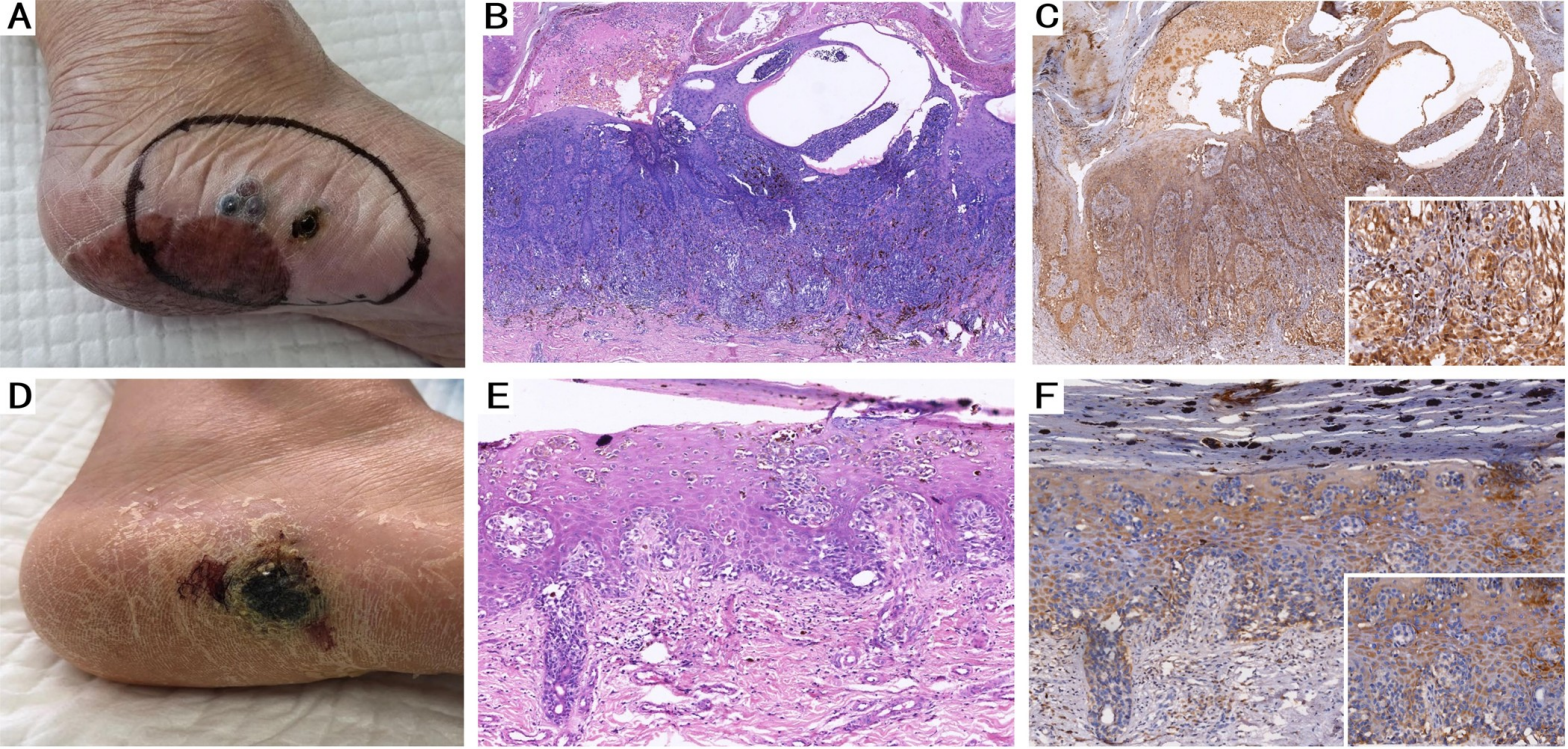
HSP105 expression in recurrent lesion and primary lesion. (A) Clinical picture of the ALM located in foot (recurrent lesion). (B) Histological appearance (HE, ×100) of ALM in recurrence lesion and (C) HSP 105 expression showed strong staining (IHC×100). (D) Clinical picture of the ALM located in foot (primary lesion). (E) Histological appearance (HE, ×100) of ALM in primary lesion and (F) HSP 105 expression showed weaker staining (IHC×100).

Next, we evaluated the clinical data of patients to determine the significance of HSP105 expression in CMM. The correlations between HSP105 immunostaining and tumor clinicopathological features are summarized in Table 2. In different clinical variants (including tumor location, duration of the lesion, metastasis and recurrence), lesions of 58 cases of CMM were located on non-exposed sites, while the lesions of 33 cases were located on exposed sites. We found that HSP105 expression was increased in exposed sites (HSCORE = 3.02 (2,70, 3.65)) compared with non-exposed sites (HSCORE = 2.48 (1.60, 3.50)) (P = 0.011) (Fig 2). In most cases, the onset time was less than 10 years (70 cases), while cases with an onset greater than 10 years were relatively rare. The relative expression levels of HSP105 in CMM were not significantly correlated with the duration of CMM lesions (P>0.05), but HSCORE values for patients with durations between 1 and 10 years (HSCORE = 2.76 (1.80, 3.80)) were relatively higher. The number of cases of metastasis and recurrence in CMM patients was smaller (27 cases) than that of primary tumors (64 cases), but we found that recurrent and metastatic CMM tumor lesions (HSCORE = 3.20 (2.70, 3.80)) exhibited higher HSP105 expression than primary tumors (HSCORE = 2.46 (1.65, 3.35)) (P = 0.001) (Fig 3).

**Table 2:** HSP105 expression in different clinicopathological features of CMM.

Variables	Number (%) N (%)	HSCOR *M* (*P*_25_, *P*_75_)	Z/X^2^/ρ	*P*
**Location**			-2.550	0.011
Exposed site	33(37.3)	3.02(2,70, 3.65)		
Non-exposed site	58(58.7)	2.48(1.60, 3.50)		
**Duration of the lesion (years)**			-0.077	>0.05
<1	31(34.1)	2.66 (1.80, 3.60)		
1–10	39(42.9))	2.76 (1.80, 3.80)		
>10	21(23.0)	2.57(1.80, 3.60)		
**Metastasis and Recurrence**			-3.336	0.001
Yes	27(29.7)	3.20 (2.70, 3.80)		
No	64(70.3)	2.46 (1.65, 3.35)		
**Pathological Classification**			17.681	0.001
LMM	17 (18.7)	3.12 (2.70, 3.80)		
NM	24 (26.4)	3.18 (2.70, 3.90)		
SSM	21 (23.1)	2.26 (1.80, 2.70)		
ALM	29 (31.8)	2.31 (1.60, 3.30)		
**Clark’s level**			0.204	0.052
1	20(22.0)	2.54 (1.80, 3.60)		
2	22(24.2)	2.48 (1.60, 3.45)		
3	24(26.4)	2.71 1.80, 3.80)		
4	17(18.7)	2.66 (1.75, 3.60)		
5	8(8.7)	3.50 (3.10 3.92)		
**Breslow thickness**			0.196	0.64
<1	10(11.0)	2.78 (1.80, 3.60)		
1~	14(15.4)	2.21 (1.60, 2.85)		
2~	43(47.2)	2.67 (1.80, 3.65)		
≥4	24(26.4)	2.89 (2.02, 3.75)		

### The relationship between HSP105 and pathological features

**Fig 4:**
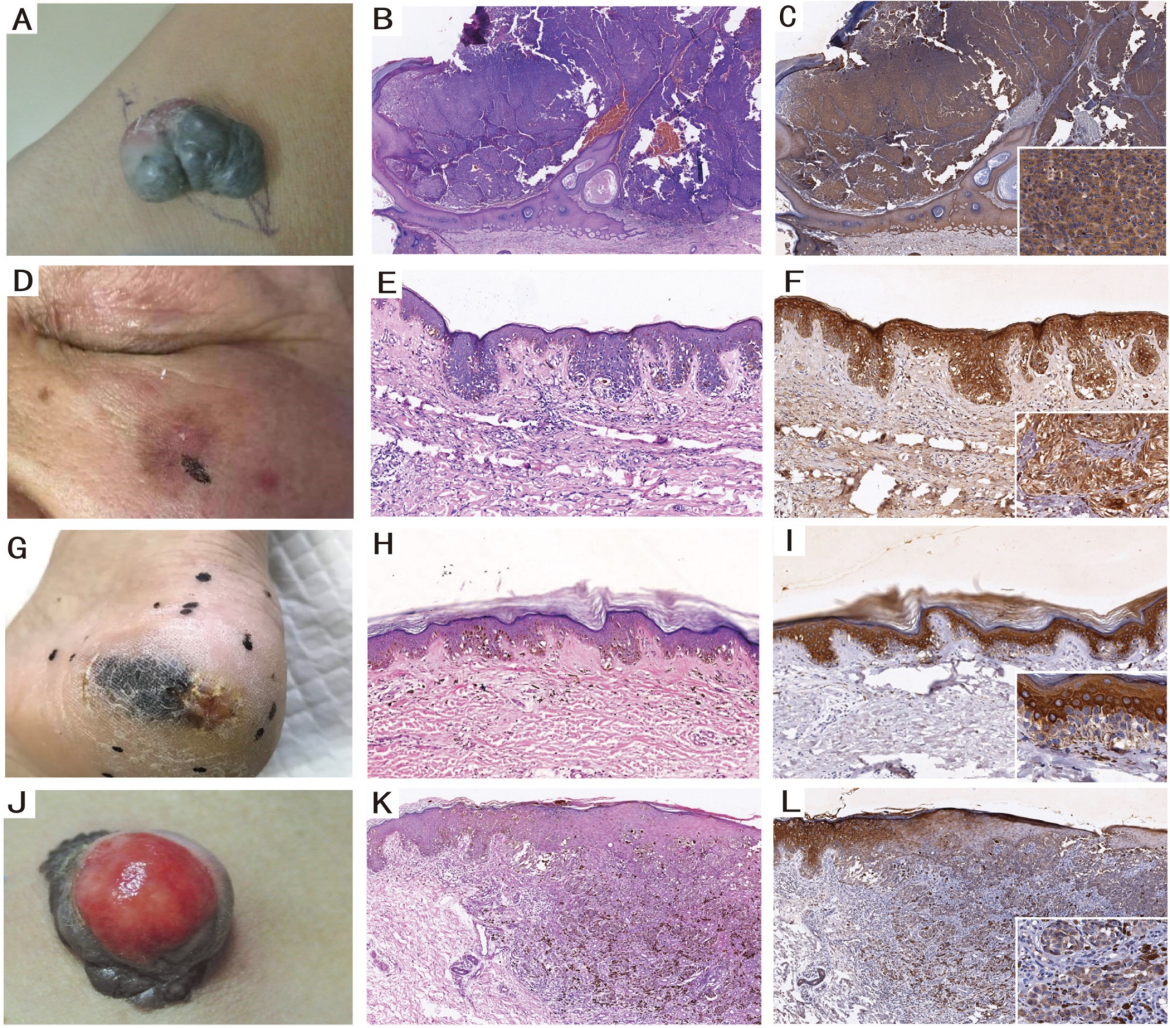
HSP105 expression in different pathological classification. (A) Clinical and (B; HE×10) histopathological picture of the NM located in wrist. (D) Clinical and (E; HE×100) histopathological picture of the LMM located in face. (G) Clinical and (H; HE×100) histopathological picture of the ALM located in foot. (J) Clinical and (K; HE×100) histopathological picture of the SSM located in thigh. HSP105 showed intense staining in (C; IHC×10) NM and (F; IHC×100) LMM, but it showed weaker staining in (I; IHC×100) ALM and (L; IHC×100) SSM.

We further detected HSP105 expression in different pathological variants (including pathological classification, Clark’s level and Breslow thickness). In different pathological classifications, the number of ALM (acral lentiginous melanoma) cases was the highest (29 cases), while NM (nodular melanoma) was observed in 24 cases, and the numbers of LMM (lentigo maligna melanoma) and SSM (superficial spreading melanoma) cases were relatively l at 17 and 21 cases, respectively. The Kruskal-Wallis test was used to show significant differences in four different pathological types of CMM (P<0.001). HSP105 expression in LMM (HSCORE = 3.12 (2.70, 3.80)) and NM (HSCORE = 3.18 (2.70, 3.90)) was higher than that in ALM (HSCORE = 1.91 (1.60, 2.10)) and SSM (HSCORE = 1.84 (1.50, 2.50)). The immunohistochemical staining intensity pattern is shown in (Fig 4). We further used the Mann-Whitney test to determine the statistical significance among the four different pathological types. Statistically significant differences were observed between LMM and ALM, LMM and SSM, NM and ALM, and NM and SSM (P<0.05). However, no significant difference was observed between the other two pathological types (P>0.05). Although lower expression of HSP105 was observed in ALM, 5 patients showed high expression in the fingernails of the hands or feet. HSP105 expression did not increase with increasing depth or grade. No statistically significant correlation was found between the HSP105 expression pattern and Clark’s level or Breslow thickness (P>0.05). However, the HSCORE values of cases with Clark 5 (HSCORE = 3.50 (3.10 3.92)) and Breslow ≥ 4 (HSCORE = 2.89 (2.02, 3.75)) were higher, which suggests that HSP105 expression may be associated with the progression of CMM.

## Discussion

HSP expression can promote controlled cell growth, avoid apoptosis, enhance cell survival, and promote angiogenesis, which is essential for the metastasis and invasiveness of many malignant cells [15]. Therefore, heat shock protein-related tumors can be referred to as "companion diseases." Previous studies have shown elevated HSP105 expression in a range of cancers, such as bladder cancer, colorectal cancer, lung cancer and oral squamous cell carcinoma, which indicates a poor prognosis in most cases [16].

In this study, we evaluated HSP105 expression in nevi and CMM; furthermore, we compared HSP105 expression in CMM with different clinicopathological features. The results revealed a remarkable difference between nevi and CMM, which was consistent with a previous study [14, 17]. Our study also revealed no difference in HSP105 expression in different pathological classifications of nevi. Exposed site lesions, NM and LMM types, recurrence and metastatic lesions were closely associated with higher HSP105 expression. Although HSP105 was expressed in these cases, no significant association was found between HSP105 expression and Clark’s level, Breslow thickness, and lesion duration.

Exposed sites had higher HSP105 expression than non-exposed sites. Previous studies have shown that heat shock can regulate UVB-induced keratinocyte death in the human epidermis [18]. Heat shock can also increase the level of heat shock proteins, which prevents UVB-induced apoptosis. HSP70 overexpression is known to ameliorate UV-induced photokeratitis induced by geranylgeranyl-lacetone in mice [19]. On the contrary, HSP70 can prevent UV-induced apoptosis in melanoma cells. Therefore, as a subtype of HSP70, HSP105 overexpression can also help prevent apoptosis in tumors, thus prompting tumor growth [20]. At the same time, chronic light injury and chemical substances are the main pathogenic factors of CMM [4], which may promote HSP105 expression at exposed sites in patients with CMM.

In terms of pathological types, HSP105 expression was lower in ALM and SMM compared with the NM and LMM types. LMM is predominantly located on the sun-exposed skin of elderly people and may promote HSP105 expression. Although NM does not account for a high proportion of all invasive melanomas, it has a poor prognosis and contributes to most melanoma-related deaths [21]. NM is usually characterized by early vertical and rapid growth followed by invasion of the dermis. The molecular chaperone heat shock proteins play an important role in maintaining the stability and activity of many signaling proteins involved in the rapid development and growth of NM. Previous studies revealed that HSP105 can prevent tumor apoptosis by inhibiting Bax translocation to mitochondria and P38 in the MAPK pathway [22]. HSP105 inhibits apoptosis and promotes continuous clonal proliferation in tumors, which may provide an environment for the rapid growth and infiltration seen in NM.

The significance of HSP105 in terms of prognosis is worth mentioning. A previous study observed that high HSP105 expression is related to metastatic lesions, which exhibit increased expression with increasing stage and thickness of the melanoma. Our study also showed significantly higher HSP105 expression in metastatic lesions but also in local recurrent lesions compared with primary cutaneous melanoma. However, no statistically significant correlation was observed between the HSP105 expression pattern and Clark’s level or Breslow thickness, the HSCORE values of cases with Clark 5 and Breslow ≥ 4 were higher. Previous studies have also revealed that HSP105 is overexpressed in a variety of human cancers; for example, the expression of HSP105 in rectal cancer in general is higher than that in rectal adenocarcinoma [23]. However, other studies have found that the prolonged survival of bladder cancer patients is dependent on higher HSP105 expression [24]. In skin tumors, basal cell carcinoma expresses weak levels of HSP105 or is negative, which is in contrast to the high expression in EMPD and metastatic SCC [25]. This study also showed that high HSP105 expression was related to recurrence and metastatic lesions, which suggests that high HSP105 expression is related to poor prognosis in CMM.

HSP105 might be a novel potential target in cancer immunotherapy since it is specifically overexpressed in various human cancers. Previous studies revealed that a HSP105 peptide vaccine could induce HSP105-specific cytotoxic T lymphocytes from peripheral blood mononuclear cells (PBMCs) at injection sites [26]. Animal studies have shown that immunization with HSP105-pulsed dendritic cells leads to tumor rejection in mice [27]. In vivo studies also showed that antitumor immunity induced by an HSP105 DNA vaccine could be used for immunotherapy or prevention of various human tumors overexpressing HSP105, including colorectal cancer and melanoma [6]. Our study may help in the development of targeted and immunotherapy treatments against HSP105 according to different clinical and pathological conditions.

This study has several limitations including a small sample size, which may have resulted in bias, and the unavailability of survival and recurrence data. Therefore, we recommend that prospective large-scale studies be performed to evaluate the prognostic and predictive significance of HSP105 in CMM. Another limitation is that the exploration of the mechanism of HSP105 in CMM is incomplete. Therefore, we recommend the establishment of cytological or animal models to analyze its pathogenic mechanism to further evaluate the correlation between HSP105 and CMM.

In conclusion, we examined the level of HSP105 expression in CMM and nevi and evaluated the relationship between HSP105 and the clinicopathological characteristics of CMM. Our study demonstrated that HSP105 is overexpressed in CMM and that higher HSP105 expression is associated with exposed site lesions, NM and LMM types, recurrence and metastatic lesions. No significant difference in HSP105 expression was observed in cases with different Clark’s level, Breslow thickness, or lesion duration.

## Supporting information

S1 Fig The western blot of HSP105 expression in skin tumors. We compared the expression of HSP105 in skin tumors, of which line 2 was CMM, line3 and 4 was nevi.
(TIF)
