# Supplementary figures and images for: HSP105 expression in cutaneous malignant melanoma: Correlation with clinicopathological characteristics
Source: PLoS One. 2021 Oct 7;16(10):e0258053. doi: 10.1371/journal.pone.0258053 (PMC8496777; doi:10.1371/journal.pone.0258053)

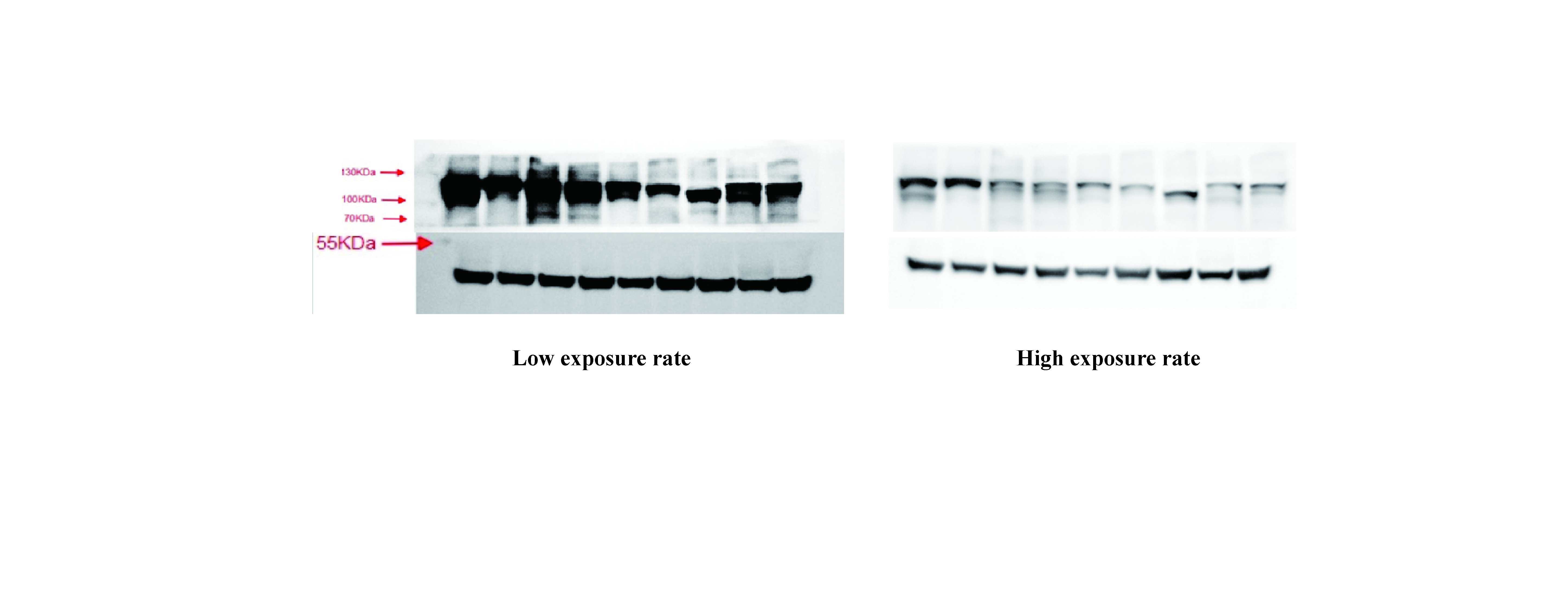

Supplement: S1 Fig — We compared the expression of HSP105 in skin tumors, of which line 2 was CMM, line3 and 4 was nevi. (TIF) [file pone.0258053.s001.tif]
